# Hsa_circ_0128846 promotes tumorigenesis of colorectal cancer by sponging hsa‐miR‐1184 and releasing AJUBA and inactivating Hippo/YAP signalling

**DOI:** 10.1111/jcmm.15590

**Published:** 2020-07-18

**Authors:** Xu Wang, Yujia Chen, Wei Liu, Tao Liu, Di Sun

**Affiliations:** ^1^ Department of Colorectal and Anal Surgery The First Hospital of Jilin University Changchun China; ^2^ Department of Gastrointestinal Surgery The First Hospital of Jilin University Changchun China; ^3^ Department of Spinal Surgery The First Hospital of Jilin University Changchun China

**Keywords:** AJUBA, circ_0128846, colorectal cancer, Hippo, miR‐1184, YAP signalling pathway

## Abstract

Hsa_circ_0128846 was found to be the most significantly up‐regulated circRNA in our bioinformatics analysis. However, the role of hsa_circ_0128846 in colorectal cancer has not been explored. We thus aim to explore the influence and mechanism of hsa_circ_0128846 in colorectal cancer by sponging its downstream miRNA target miR‐1184. We collected 40 colorectal cancer patients’ tumour tissues to analyse the expression of hsa_circ_0128846, miR‐1184 and AJUBA using qRT‐PCR and Western blot where needed. Then, we constructed stably transfected SW480 and HCT116 cells to study the influence of hsa_circ_0128846, miR‐1184 and AJUBA on colorectal cancer cell phenotypes. To obtain reliable results, a plethora of experiments including RNA immunoprecipitation assay, flow cytometry, EdU incorporation assay, wound healing migration assay, transwell invasion assay and live imaging of nude mice xenograft assay were performed. The binding relationship between hsa_circ_0128846, miR‐1184 and AJUBA mRNA in colorectal cancer was validated by reported gene assay. In colorectal cancer tissues, circ_0128846 and AJUBA were both significantly up‐regulated, while miR‐1184 was significantly down‐regulated compared with healthy tissues. Meanwhile, hsa_circ_0128846 can absorb miR‐1184 to promote the progression of CRC in vivo and SW480 and HCT116 cell phenotypes in vitro. The knockdown of AJUBA, a downstream target of miR‐1184, reversed the effect of miR‐1184 in CRC cells via enhancing the phosphorylation of the Hippo/YAP signalling pathway proteins MST1, LATS1 and YAP. This study revealed that hsa_circ_0128846 contributed to the development of CRC by decreasing the expression of miR‐1184, thereby increasing AJUBA expression and inactivating Hippo/YAP signalling.

## 1 INTRODUCTION

Colorectal cancer (CRC) is not only the world's third most usual disease, but also the second most death‐leading cancer‐related disease.^1^ Clinically, distant metastasis is a preponderant justification of death in CRC that can affect the 5‐year survival time and the quality of life of the patients.^2^ Given the poor survival time and life quality of patients, it is meaningful to discover new treatments for CRC. Many researches have been reported that molecular targeted therapy has become a new treatment for CRC besides conventional surgery and chemoradiotherapy.^3^, ^4^, ^5^ At present, some molecular targeted therapies have come out such as anti‐VEG monoclonal antibody, VEGF tyrosine kinase inhibitor and anti‐EGFR monoclonal antibody which can improve patient's quality of life.^6^, ^7^, ^8^, ^9^, ^10^ Nevertheless, little therapeutic effects happen in CRC patients taking the molecular targeted therapy drugs. Hence, it is imperative to find more effective therapeutic targets to treat CRC.

Circular RNA (circRNA) is a newly discovered non‐coding RNA with a circular structure, which is different from other RNAs.^11^, ^12^ CircRNAs are stable in the cytoplasm, which means that they are not easy to be degraded.^11^, ^12^, ^13^, ^14^ In recent researches, circRNAs have been found to have affluent binding sites for microRNAs.^11^, ^12^, ^15^, ^16^ By binding with circRNAs, miRNAs thus release their target mRNAs.^11^, ^12^, ^15^, ^16^ This mechanism of action was known as the competitive endogenous RNA (ceRNA) network.^11^, ^12^, ^15^, ^16^ It has been reported that circRNAs could affect the development of colorectal cancer through the ceRNA network mechanism.^17^, ^18^, ^19^ However, there has been no research reporting circ_0128846 as a CRC development regulator.

MiR‐1184 has been reported to be down‐regulated in various tumours such as bladder cancer, breast cancer and colorectal cancer, and up‐regulated in non‐small‐cell lung cancer and prostate cancer.^20^, ^21^, ^22^, ^23^, ^24^, ^25^ It was once reported by Dengke Yang *et al* that miR‐1184 got absorbed by circVANGL1 thus enhancing the bladder cancer phenotypes.^20^ In this study, a novel ceRNA network involving miR‐1184 and its upstream regulator, hsa_circ_0128846, is to be unravelled in CRC.

AJUBA protein is a member of the LIM protein subfamily with three tandem LIM domains at the C‐terminus.^26^ AJUBA can be transferred between the cytoplasm and the nucleus due to its nuclear importation and nuclear exportation sequences.^27^, ^28^, ^29^ AJUBA has been proved to regulate the transmission of signals from the cytoplasm to the nucleus, and to participate in many signal transducer interactions such as JAK/SATA, Hippo/YAP, Smad/Snail and Wnt/β‐catenin.^29^, ^30^, ^31^, ^32^, ^33^ AJUBA was once reported to be up‐regulated in CRC,^34^ and to promote CRC cell survival,^30^ suggesting that it is a possible regulator in CRC. Also, it has been reported that AJUBA could be regulated by miRNAs.^35^, ^36^ Nonetheless, how AJUBA being regulated by miRNAs in CRC has not been studied.

In our study, we first determined the stimulating effects of hsa_circ_0128846 on the development of CRC via in vivo and in vitro experiments. We also found that circ_0128846 could sponge miR‐1184 to elevate the expression level of AJUBA for accelerating the progression of CRC. Besides, the regulative mechanism of hsa_circ_0128846/miR‐1184/AJUBA ceRNA network on CRC might be related to the Hippo/YAP signalling pathway. Our findings may exhibit a new target for the treatment of CRC.

## 2 MATERIALS AND METHODS

### 2.1 Patients and cell lines

CRC tissues (n = 40) and adjacent healthy colon tissues (n = 24) collected from CRC patients from the First Hospital of Jilin University were used in this study. The collection and the use of tissues followed by the ethical standards in the Helsinki Declaration. The informed consent was signed by all patients. The clinical characteristics are shown in Table 1. The study protocol was approved by the ethics committee of the First Hospital of Jilin University.

**Table 1:** Clinical parameters of patients with colorectal cancer in this study

Pathological characteristics	Case(n)
*Gender*	
Male	24 (60%)
Female	16 (40%)
*Age*	
≥25	22 (55%)
＜25	18 (45%)
*Tumour differentiation*	
Well/moderately	25 (62.5%)
Poorly	15 (37.5%)
*TNM stages*	
I‐II	19 (47.5%)
III‐IV	21 (52.5%)
*Tumour size*	
<5 cm	22 (55%)
≥5 cm	18 (45%)
*Distant metastasis*	
Negative	18 (45%)
Positive	22 (55%)

### 2.2 Real‐time quantification PCR

Total RNA from tissue samples and cells was dissociating by kit from Tiangen Biochemical (DP501, China) as well as RNA reverse transcription. Before we performed the RNA reverse transcription, we used gel electrophoresis to check the purity of the RNA. Then, the instrument of 7500 from ABI was used to analyse the expression of circ_0128846, miR‐1184 and mRNA of AJUBA in CRC tissues and CRC cells with using SYBR Green PCR Kit (Takara, RR820A, Japan). GAPDH was used as the reference gene for circ_0128846 and AJUBA, and U6 was used as the reference miRNA for miR‐1184. All the primers were purchased from GeneCopoeia (Guangzhou, China), and the sequences of primers are shown in Table 2.

**Table 2:** The sequences of the primers in this study

Primer	Sequences
*Hsa_circ_0128846*	
Forward sequence	5′‐GACCTCTGTCAGCGAGTTCC‐3′
Reverse sequence	5′‐GCTACTGGAGCCTGATGGAC‐3′
*miR‐1184*	
Forward sequence	5′‐CCTGCAGCGACTTGATGGC‐3′
Reverse sequence	5′‐GAACATGTCTGCGTATCTC‐3′
*AJUBA*	
Forward sequence	5′‐GCTGCTGTTTCCCTCTGGAT‐3′
Reverse sequence	5′‐CCCTCCTGTGTGTGCCTAAG‐3′
*GAPDH*	
Forward sequence	5′‐GTCAAGGCTGAGAACGGGAA‐3′
Reverse sequence	5′‐AAATGAGCCCCAGCCTTCTC‐3′
*U6*	
Forward sequence	5′‐TGCGGGTGCTCGCTTCGGCAGC‐3′
Reverse sequence	5′‐CCAGTGCAGGGTCCGAGGT‐3′

### 2.3 Cell culture

The human CRC cell lines (HCT116, SW480, CW‐2, RKO and DLD‐1) were purchased from Cell Resource Center, Institute of Basic Medical Sciences, Chinese Academy of Medical Sciences (China). The human normal colon cell line (HCoEpiC) was purchased from BNCC (China). HCT116 cells were cultured in McCoy's 5A Media which had supplemented with 10% FBS, and the cells were humidified incubator with 5% CO2 at 37°C. HCoEpiC cells were cultured in DMEM, while SW480, CW‐2, RKO and DLD‐1 cells were cultured in RPMI 1640 with other condition same with HCT‐116.

### 2.4 RNase R treatment

Total RNA was digested by RNase R (Tiangen Biochemical, China) in 37°C for 15 minutes with 40U. Next, equal amount of RNA was reverse‐transcribed into cDNA. The expression of circ_0128846 in cDNA was analysed by qRT‐PCR. The expression level is positively correlated with the stability of hsa_circ_0128846 and its linear counterpart, ZFR mRNA.

### 2.5 Constructs of circ_0128846 shRNA lentiviral vectors and circ_0128846 overexpression vectors

The pLV‐CMV‐puro‐U6‐shRNA lentiviral construct was purchased from OBiO Technology Shanghai Corp., Ltd. (China) and was used to establish the construct that could stably silence circ_0128846. The construction of pLV‐CMV‐puro‐U6‐sh‐circ_0128846 lentiviral vector (sh‐circ_0128846) was based on a previous study.^37^ Briefly, a total of three pairs of shRNA sequences for silencing hsa_circ_0128846 were designed against the joint site of hsa_circ_0128846, and the corresponding sequences of these shRNAs are shown in Table 3. Then, the three shRNAs of hsa_circ_0128846 were synthesized and inserted between the EcoRI and XhoI sites of the pLV‐CMV‐puro‐U6‐shRNA lentiviral vector using the corresponding restriction enzymes (Thermo Fisher Scientific, USA). As for hsa_circ_0128846 overexpression vectors, the pLCDH‐circ‐circ_0128846 overexpression vector (OE‐circ_0128846) obtained from GeneCopoeia (Guangzhou, China) was used to transfect cells. Briefly, the full‐length cDNA of hsa_circ_0128846 was amplified by PCR using PrimerSTAR Max DNA Polymerase Mix (TaKaRa, Japan). Then, the cDNA of hsa_circ_0128846 was inserted into the overexpression vector, pLCDH‐cir (GenePharma, China). Puromycin was used to screen out the successfully transfected cells. To identify the transfection efficiency of sh‐circ_0128846 and OE‐circ_0128846, qRT‐PCR was conducted to detect the expression of hsa_circ_0128846 and its linear counterpart, ZFR mRNA, after the cells were transfected with pLV‐CMV‐puro‐U6‐sh‐circ_0128846 vectors or pLCDH‐circ‐circ_0128846 overexpression vector for 48 hours.

**Table 3:** The oligonucleotide sequences of the shRNAs in the loss‐of‐function experiments

Primer	Oligonucleotide sequences
*shRNA1‐circ_0128846*	
Forward sequence	5′‐CAACUACAGUUGCUAGCUACA‐3′
Reverse sequence	5′‐UAGCUAGCAACUGUAGUUGGA‐3′
*shRNA2‐circ_0128846*	
Forward sequence	5′‐GUCCUGUGUGAUUAUCAUACG‐3′
Reverse sequence	5′‐UAUGAUAAUCACACAGGACUG‐3′
*shRNA3‐circ_0128846*	
Forward sequence	5′‐GCCUAUUCUCAUCCAACUACA‐3′
Reverse sequence	5′‐UAGUUGGAUGAGAAUAGGCUA‐3′
*shRNA1‐AJUBA*	
Forward sequence	5′‐GCACCTGTATCAAGTGCAA‐3′
Reverse sequence	5′‐TTGCACTTGATACAGGTGC‐3′
*shRNA2‐AJUBA*	
Forward sequence	5′‐GGAGCGGTTAGGAGAGAAA‐3′
Reverse sequence	5′‐TTTCTCTCCTAACCGCTCC‐3′
*shRNA3‐AJUBA*	
Forward sequence	5′‐GGACCTAGCTTTAAATCAA‐3′
Reverse sequence	5′‐TTGATTTAAAGCTAGGTCC‐3′

### 2.6 Other constructs and cell transfection

miR‐1184 inhibitor and negative control plasmids were provided by GenePharma (Shanghai, China). The AJUBA lentiviral construct (pLV‐CMV‐puro‐U6‐sh‐AJUBA) was provided by OBiO Technology Shanghai Corp., Ltd. (China). Briefly, a total of three shRNAs of AJUBA were designed and inserted to the pLV‐CMV‐puro‐U6‐shRNA lentiviral vector in the same way as sh‐circ_0128846. The sequences of sh‐AJUBA are shown in Table 3. Then, the constructs mentioned above were transfected into the SW480 and HCT116 cells for 48 hours using Lipofectamine 2000 reagent (Thermo Fisher, 11668027, USA) on the basis of the protocol given in the manual. The cells in the blank group were cultured without any transfection. The transfection efficiency was assessed by qRT‐PCR.

### 2.7 EdU assay

EdU Staining Proliferation Kit was obtained from Solarbio (China) for the assessment of cell proliferation capability. Briefly, the 1 × 10^5^ transfected SW480 and HCT116 cells were seeded in the 96‐well plate. After an incubation for 48 hours, 50 mM EdU was added to each well to continue to incubate cells for 2 hours. Then, the cells were fixed by 4% formaldehyde for 30 minutes, stained by 100 μL Apollo reagent for 30 minutes and finally stained by 10 μL DAPI reagent for 15 minutes. The representative images of EdU assay were taken using a fluorescence microscope (Nikon, Japan).

### 2.8 The detection of cell apoptosis and cell cycle

Flow cytometry was used to measure the cell apoptosis and cell cycle. To detect cell apoptosis, we used 1× binding buffer including Annexin V‐FITC to suspend the transfect CRC cells and incubated at 37°C for 10 minutes in the dark after washing the cell suspension by PBS for three times. Before we analysed by FACScan flow cytometer, the cells were stained with 5 μL propidium iodide. For cell cycle, the transfected SW480 and HCT116 cells were collected and suspended at a density of 3 × 10^4^/mL. The cell suspension was then rinsed by PBS for three times and fixed with pre‐chilled methanol at 4°C for 4 hours. Subsequently, PBS with 50 μg/mL propidium iodide, 100 μg/mL RNase A and 0.2% Triton X‐100 was used to treat the cell suspension in the dark for 30 minutes. Lastly, the cell cycle of cells in every group was profiled using the FACS flow cytometer.

### 2.9 Cell wound healing migration and cell transwell invasion assay

The 4 × 10^5^ cells were seeded into the 12‐well plate before we scratched with the 20 μL micropipette tip in the centre of the cell monolayer. Then, the remaining cells were cultured with medium that contained no serum. At 0 and 24 hours, we captured images to observe the result of wound healing. In invasion assay, we seeded cells into the upper chambers precoated with Matrigel of the transwell at the density of 1 × 10^5^/mL. The medium in upper chambers was serum‐free, while the serum concentration in the medium of the lower chambers was 10%. After 24 hours, we washed the transwell membrane by PBS, fixed the membrane in 4% methanol for 15 minutes and stained the cells attached to the membrane in 0.1% crystal violet for 15 minutes. Finally, we counted the number of cells that invaded through the transwell membrane in randomly selected three fields under a microscope.

### 2.10 Animal studies

The animal experiment was carried out following the guidelines of the Institutional Animal Care and Use Committee (IACUC). Six BALB/c nude mice were purchased from Beijing Vital River Laboratory Animal Technology Co., Ltd. The nude mice were divided into two groups (NC and sh‐circ_0128846). Then, the SW480 cells transfected with sh‐circ_0128846 or NC were injected into the nude mice in the corresponding groups. The tumour volume was measured every six days. After 30 days, the IVIS 200 bioluminescence imaging system and Living Image software (Caliper Life Sciences, Hopkinton, MA) were used to monitor and analyse tumour growth. The nude mice were killed after 30 days to collect the xenografted tumours for the following Ki67 and H&E staining.

### 2.11 Ki67 and H&E staining

First, tumours collected from the animal experiments were fixed in 4% formalin for 48 hours. Then, the tissues were sequentially placed in 75%, 80%, 95% ethanol and absolute ethanol to dehydrate. After dehydration, the tissues were dipped in melted paraffin, and then embedded in an embedder. Next, we used a tissue slicer to slice tumours into sections in 5 μm thickness. Before we started staining, we dewaxed the sections. Then, we treated the sections with peroxide blocker to remove endogenous peroxidases. Finally, the sections were incubated with the anti‐Ki67 primaries (Cat#: ab16667, Abcam, UK) and the HRP‐conjugated rabbit secondaries (Cat#: 6728, Abcam, UK) after being washed in PBS. In H&E staining, after dewaxing the sections, we stained the sections with haematoxylin for 5 minutes and eosin for 20 seconds.

### 2.12 Reporter gene assay

Wild‐type strains and mutants of hsa_circ_0128846 and AJUBA were constructed by GeneCopoeia (Guangzhou, China). Briefly, the wild‐type fragments of hsa_circ_0128846 and AJUBA containing the binding site of miR‐1184 were amplified by PCR and used to construct the wild‐type plasmids. To construct mutants, the fragments of hsa_circ_0128846 and AJUBA with the binding site for miR‐1184 were mutated using QuikChange XL Site‐Directed Mutagenesis Kit (Agilent Technologies, USA). Next, the wild‐type strains or mutants of hsa_circ_0128846 and AJUBA were inserted into the downstream of pGL3 luciferase vectors (Promega, USA) between HindIII and Xhol restriction sites. Finally, the pGL3‐circ_0128846‐WT, pGL3‐circ_0128846‐MUT, pGL3‐AJUBA‐WT or pGL3‐AJUBA‐MUT plasmids were transfected into HCT116 and SW480 cells together with miR‐1184 mimics or miR‐1184 negative controls (NC) using Lipofectamine 2000 reagent (Thermo Fisher, 11668027, USA). 48 hours later, the cells were lysed in lysis buffer. The Promega system was employed for the detection of the luciferase activity.

### 2.13 Western blot assay

The cell protein was obtained using RIPA buffer that included 5 mM EDTA and PMSF. All protein samples were quantified using a spectrophotometer to ensure that the protein samples were the same concentration. The protein samples were then separated using sodium dodecyl sulphate‐polyacrylamide gel electrophoresis and transferred onto PVDF membranes (Millipore, MA, USA), which were incubated with primary antibodies against AJUBA(Cat#: ab244285, Abcam, UK), phospho‐MST1 (Cat#: 49332, Cell Signaling Technology, USA), MST1 (Cat#: 3682, Cell Signaling Technology, USA), phospho‐LATS1 (Cat#: 9159, Cell Signaling Technology, USA), LATS1 (Cat#: 3477, Cell Signaling Technology, USA), phospho‐YAP (Cat#: ab62751, Abcam, UK), YAP (Cat#: ab81183, Abcam, UK) and GAPDH (Cat#: ab8245, Abcam, UK) for four hours, respectively. GAPDH was used as an internal loading control. Then, the hybrid membranes were incubated with secondaries for 2 hour. The membrane went through exposure, and the protein band intensity was read using FluorChem FC2 software.

### 2.14 RNA immunoprecipitation (RIP) assay

RIP assay was performed to validate the binding relationship between has‐circ_0128846 and miR‐1184 using Magna RIP RNA‐Binding Protein Immunoprecipitation Kit (Millipore, 17‐700, USA) in accordance with the protocols. SW480 and HCT116 cells that transfected with miR‐1184 mimic or NC were lysed in RIP lysis buffer containing magnetic beads conjugated with human anti‐AGO2 antibodies or IgGs. Then, Proteinase K was added to the mixture to remove the proteins. Lastly, the immunoprecipitated RNA was collected, and the abundance of hsa_circ_0128846 was detected by qRT‐PCR.

### 2.15 Statistical analyses

We did the statistical analysis using GraphPad Prism v7.0. All results came from at least three independent experiments. Data were expressed as mean ± SD. Two‐tailed t test analysis was conducted for analysing differences between two groups, while one‐way ANOVA with Dunnett's multiple comparison method was conducted for analysing differences among multiple groups. *P*‐values that was less than 0.05 were deemed as statistically significant.

## 3 RESULTS

### 3.1 Expression, characterization and subcellular distribution of circ_0128846 in CRC tissue samples and cell lines

**Figure 1:**
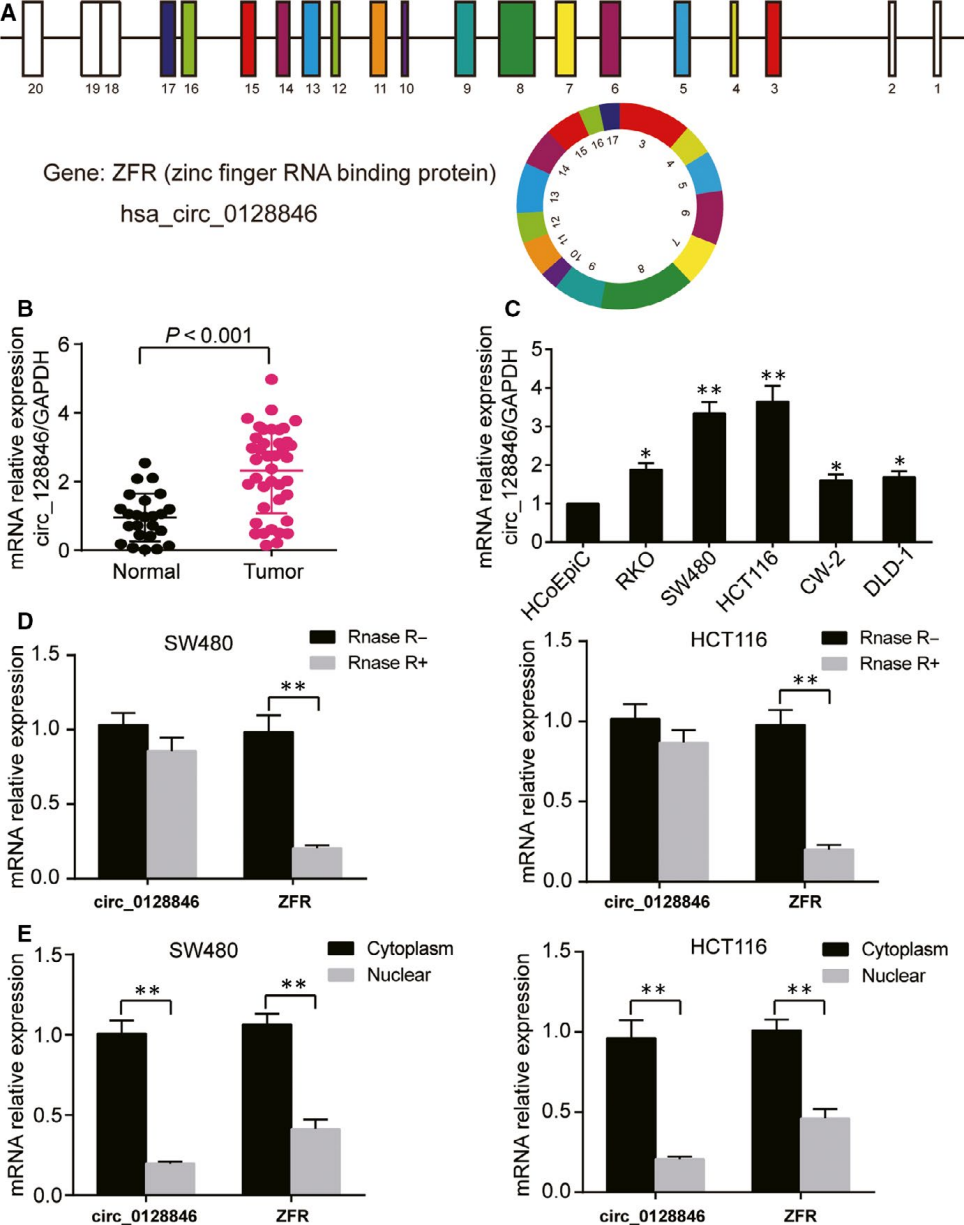
Hsa_circ_0128846 was highly expressed in CRC tissue and cells. (A) The structure of hsa_circ_0128846. (B) The qRT‐PCR was used to detect the expression of hsa_circ_0128846 in CRC tissues and adjacent healthy tissues. (C) The expression of hsa_circ_0128846 was measured in CRC cell lines (RKO, SW480, HCT116, CW‐2, DLD‐1) and human normal colon cell line (HCoEpiC). *P < .05, **P < .001 versus HCoEpiC cells. (D) The stability of hsa_circ_0128846 was detected by qRT‐PCR. RNase R was used to digest hsa_circ_0128846 and mRNA of its host gene ZFR. *P < .05, **P < .001. (E) The distribution of hsa_circ_0128846 and ZFR mRNA was identified by qRT‐PCR in cell nucleus and cytoplasm. *P < .05, **P < .001

The hsa_circ_0128846 was generated from ZFR gene. It was identified to be the most significant up‐regulated circRNA by our bioinformatics analysis (Fig. S1A). Its structure is shown in Figure 1A. Firstly, we employed qRT‐PCR to explore the expression of hsa_circ_0128846 in CRC tissues and adjacent normal tissues, and found that the expression of hsa_circ_0128846 in CRC tissues was 2.2‐fold higher than that in the normal tissues (Figure 1B). In addition, qRT‐PCR was also conducted to measure the expression of hsa_circ_0128846 in CRC cells and normal colorectal cells, and the data showed that the expression of hsa_circ_0128846 was significantly up‐regulated in CRC cells in comparison with the normal colorectal cells, especially in SW480 and HCT116 cells (Figure 1C). Therefore, SW480 and HCT116 cell lines were selected as our cell line models. Then, RNase R degradation assay was used to confirm the stable existence of hsa_circ_0128846 in CRC cells. The data demonstrated that the hsa_circ_0128846 resisted to the RNase R treatment, while the linear RNA (ZFR mRNA) was degraded in both SW480 and HCT116 cells (Figure 1D). Lastly, qRT‐PCR was performed to, respectively, measure the expression of hsa_circ_0128846 in cytoplasm and nuclear, and it was confirmed that hsa_circ_0128846 majorly existed in the cytoplasm of SW480 and HCT116 cells (Figure 1E).

### 3.2 Hsa_circ_0128846 knockdown impeded the CRC cell phenotype, whereas hsa_circ_0128846 overexpression enhanced it in vitro

**Figure 2:**
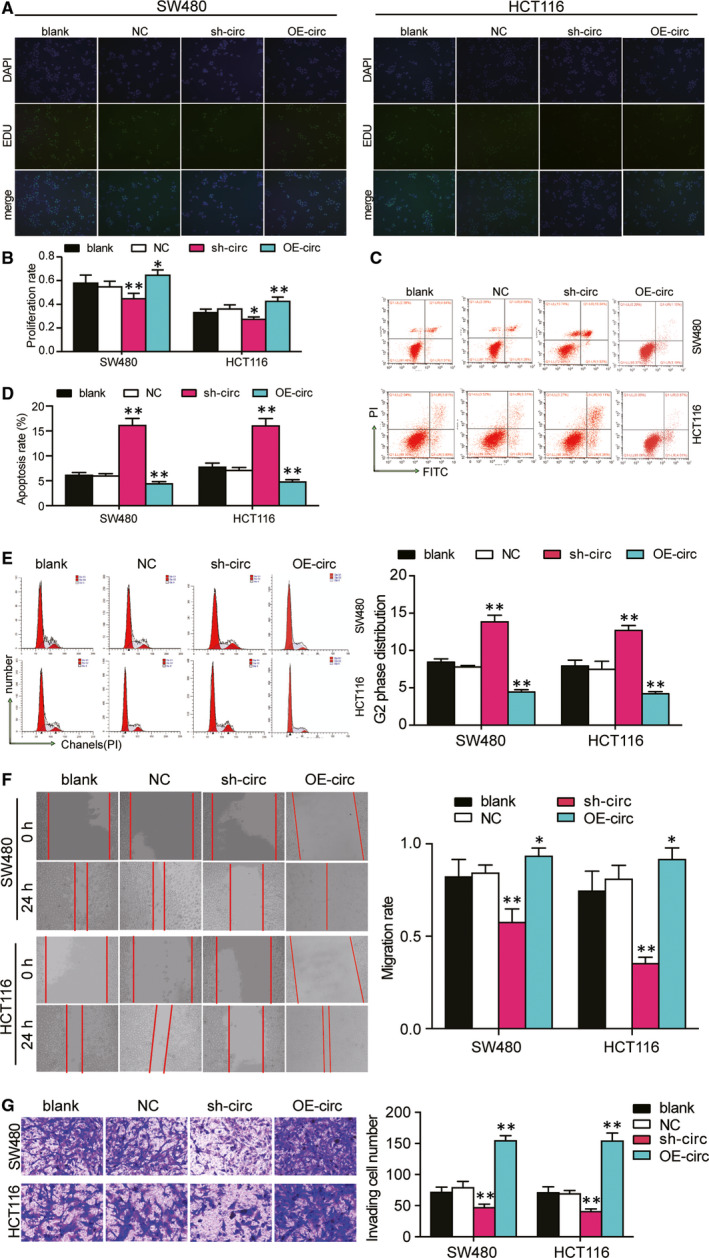
Hsa_circ_0128846 promoted the development of CRC in vitro. (A) EdU assay was used to assess the proliferation of the transfected SW480 and HCT116 cells. The merged images were from EdU staining (green) and DAPI (blue) for SW480 and HCT116 cells. (B) The proliferation rate was measured and analysed based on the EdU assay. (C) The cell apoptosis was detected by flow cytometry in transfected SW480 and HCT116 cells. The upper right (UR) quadrant: the late apoptosis. The lower right (LR) quadrant: the early apoptosis. (D) The sum of the UR and LR was calculated as the apoptosis rate. (E) Flow cytometry was performed to identify the cell cycle progression in the transfected SW480 and HCT116 cells. (F) The wound healing assay was used to assess the migration ability of SW480 and HCT116 cells after transfection at 0 and 24 hours. (G) The number of invaded cells was identified by transwell invasion assay. Blank, the cells were cultured without any treatments. NC, the cells were transfected with negative control plasmids. sh‐circ, the cells were transfected with circ_0128846 shRNA vectors. OE‐circ: the cells were transfected with circ_0128846 overexpression vectors *P < .05, **P < .001 versus blank group

We conducted loss‐of‐function and gain‐of‐function experiments to determine the effect of hsa_circ_0128846 on CRC. Before the experiments, we detected the transfection efficiency of the sh‐circ_0128846 and hsa_circ_0128846 overexpression vectors in SW480 and HCT116 cells. By qRT‐PCR, shRNA1‐circ_0128846 most efficiently reduced the expression of hsa_circ_0128846, by 70% (Fig. S2A), but did not affect the expression of linear RNA of ZFR (Fig. S2B). Therefore, shRNA1‐circ_0128846 was selected to knock down hsa_circ_0128846. In addition, the hsa_circ_0128846 overexpression vectors were successfully transfected into the SW480 and HCT116 cells as well, seen by the approximately 2‐fold increase of hsa_circ_0128846 expression (Fig. S2C). The EdU assay was then used to assess the cell proliferation capability in the CRC cells transfected with sh‐circ_0128846. The result showed that the introduction of sh‐circ_0128846 induced more than 30% decrease of cell proliferation rate in the SW480 cells and almost 20% decrease of cell proliferation rate in HCT116 cells compared with the blank group, while the introduction of OE‐circ_0128846 led to 1.12‐fold increase of cell proliferation rate in the SW480 cells and 1.33‐fold increase of cell proliferation rate in HCT116 cells compared with the blank group (Figure 2A and B). On the contrary, the apoptosis rate increased by approximately 3‐fold in the sh‐circ_0128846 group compared with the blank group, but decreased by approximately 30% in the OE‐circ_0128846 group compared with the blank group in SW480 and HCT116 cells (Figure 2C and D). The cell cycle of SW480 and HCT116 cells was arrested at G2 phase when the SW480 and HCT116 cells were transfected with sh‐circ_0128846, and the cell cycle progression was accelerated when the CRC cells were transfected with OE‐circ_0128846 (Figure 2E). The cell migration rate was measured by wound healing assay. It was displayed that the cell migration rate of the sh‐circ_0128846 group was reduced by 25% in the SW480 cells and by 57% in the HCT116 cells compared with the blank control. The cell migration rate of the OE‐circ_0128846 group was elevated by approximately 1.2‐fold in CRC cells compared with the blank group (Figure 2F). The result of cell invasion displayed a similar trend with that of cell migration (Figure 2G). These data indicated that hsa_circ_0128846 was a tumour promoter for the development of CRC in vitro.

### 3.3 Hsa_circ_0128846 promoted the CRC tumour development in vivo

**Figure 3:**
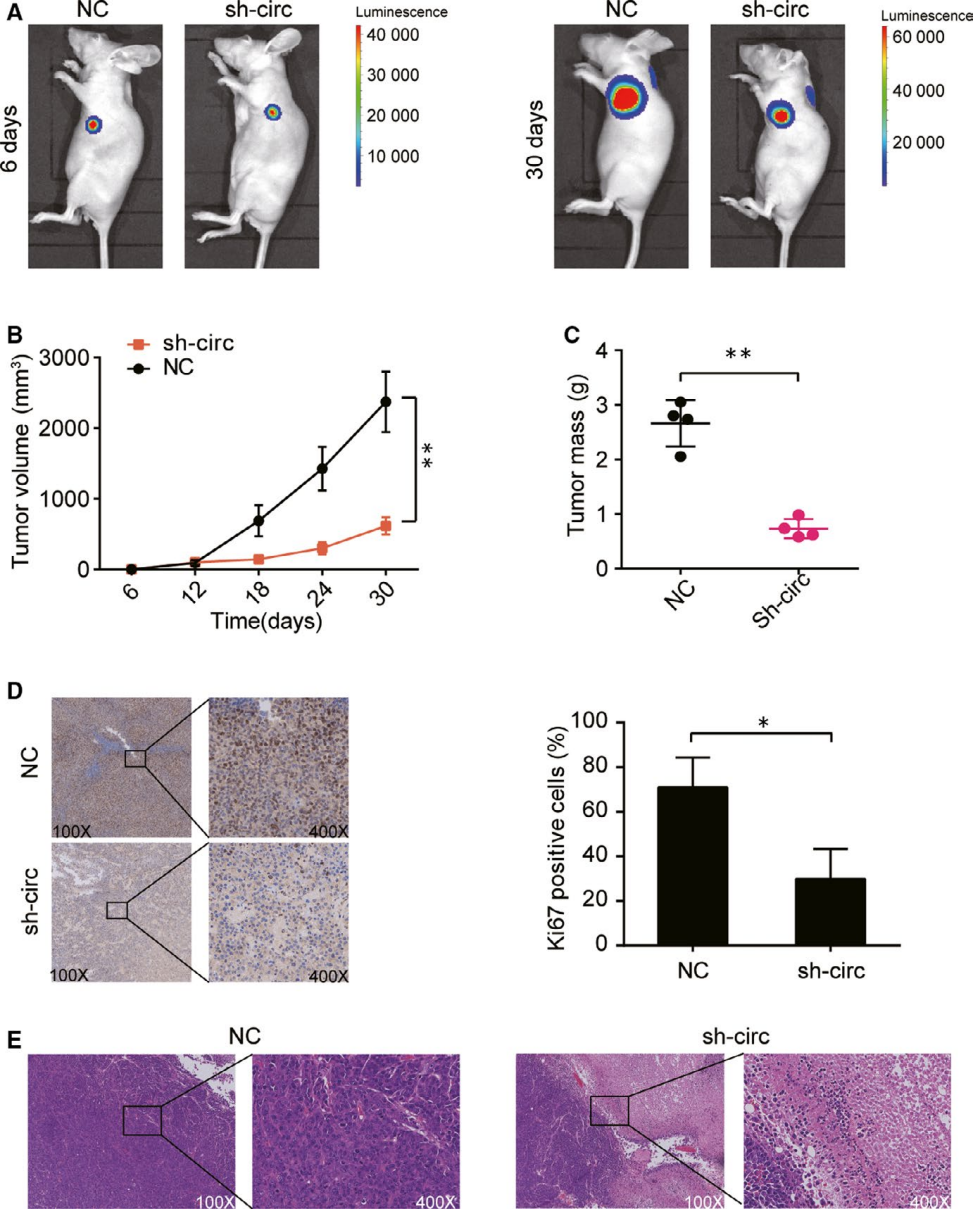
Silencing circ_0128846 inhibited the tumorigenesis of CRC cells in vivo. (A) Bioluminescent images from the nude mice injected with the SW480 cells with stable sh‐circ 0128846 or NC. (B) The tumour volume was measured every 6 days. (C) The tumour mass was measured after 30 days. (D) The representative immunohistochemistry images of Ki67 in the tumour tissues of nude mice injected by the SW480 cells with sh‐circ 0128846 or NC. (E) The representative H&E staining images from the nude mice injected with stably transfected SW480 cells. NC, the nude mice were injected with the SW480 cells with negative control plasmids. sh‐circ, the nude mice were injected with the SW480 cells stably transfected with circ_0128846 shRNA plasmids. *P < .05, **P < .001, compared with the NC group

To further ascertain the CRC promoting role of hsa_circ_0128846, we conducted in vivo animal experiments. By implanting SW480 cells stably transfected with sh‐circ_0128846 or sh‐NC vectors into the abdominal cavity of the nude mice, we established the CRC animal models. We found that mice in the sh‐circ_0128846 group showed stronger luminescence signals than those in the sh‐NC group (Figure 3A). The tumour volume of mice in sh‐circ_0128846 group was significantly smaller than that of mice in the sh‐NC group at days 18, 24 and 30 (Figure 3B). The tumour in mice of sh‐circ_0128846 was also found significantly lighter than in mice of the sh‐NC group at day 30 when the mice went through euthanasia (Figure 3C). Moreover, Ki67 staining results showed that the knockdown of circ_0128846 caused repression of Ki67 protein expression in tumour tissues (Figure 3D), suggesting that circ_0128846 silence inhibited the tumour growth. H&E staining results showed that the knockdown of circ_0128846 sabotaged the pathological structure of the tumour in nude mice (Figure 3E). These data suggested that hsa_circ_0128846 could enhance CRC tumour growth in vivo.

### 3.4 Hsa‐miR‐1184 was a downstream target of hsa_circ_0128846

**Figure 4:**
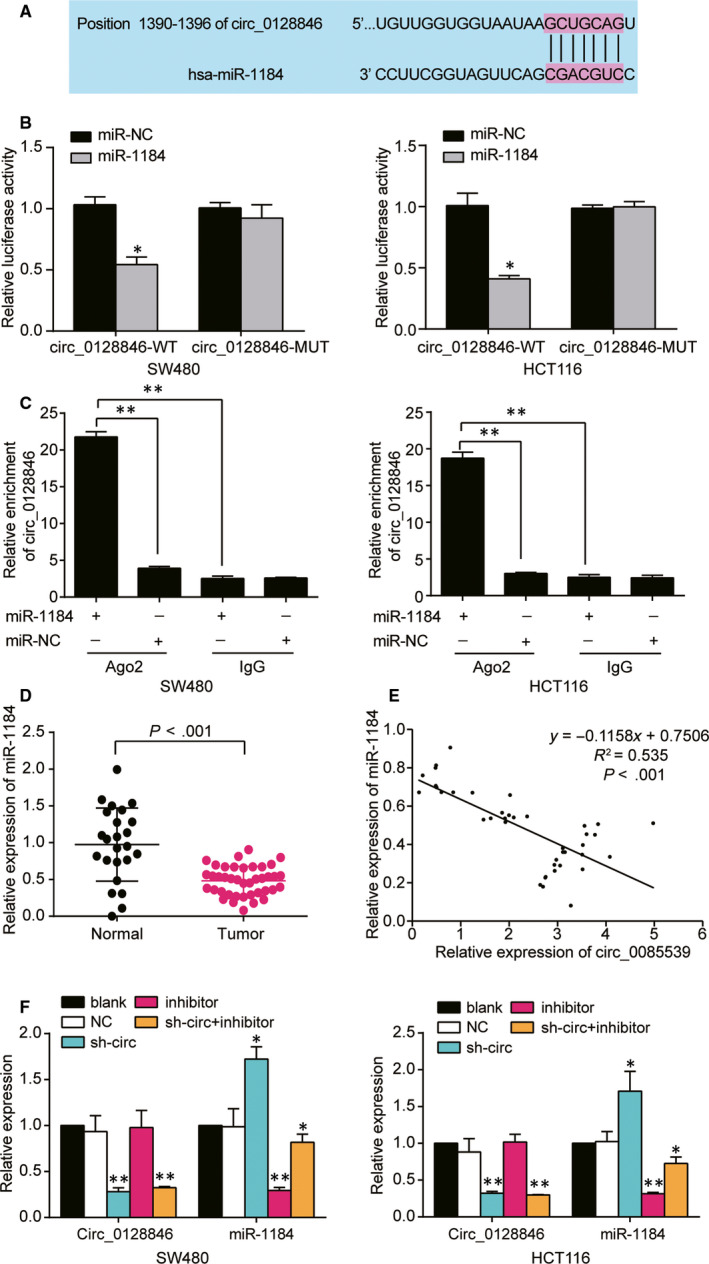
Hsa_circ_0128846 directly targeted miR‐1184. (A) The Circular RNA Interactome database was used to obtain the binding site between hsa_circ_0128846 and miR‐1184. (B) The luciferase reporter assay was used to validate the relationship between hsa_circ_0128846 and miR‐1184 in SW480 and HCT116 cells. *P < .05 versus the miR‐NC subgroup. (C) The hsa_circ_0128846 was proved to be a sponge for miR‐1184 by RIP assay. *P < .05, **P < .001, compared with miR‐1184‐Ago2 group. (D) The expression of miR‐1184 was down‐regulated in tumour tissues. (E) MiR‐1184 expression was negatively correlated with hsa_circ_0128846 expression. (F) qRT‐PCR was employed to determine the expression of hsa_circ_0128846 and miR‐1184 in the SW480 and HCT116 cells with different transfection. Blank, the cells were cultured without any treatments. NC, the cells were transfected with negative control plasmids. sh‐circ, the cells were transfected with circ_0128846 shRNA plasmids. Inhibitor, the cells were transfected with miR‐1184 inhibitor plasmids. sh‐circ + inhibitor, the cells were co‐transfected with circ_0128846 shRNA and miR‐1184 inhibitor plasmids. *P < .05, **P < .001 versus blank group

Among the identified three miRNAs (from the intersection of differentially expressed miRNAs of GSE126095 data, the targets of hsa_circ_0128846 from circular RNA interactome database and the targets of AJUBA mRNA from TargetScan Human 7.2 database, Fig. S1D), hsa‐miR‐1184 was the most significantly down‐regulated one, therefore being selected as our candidate miRNA of the ceRNA network. The complementary sequences of miR‐1184 and hsa_circ_0128846 were obtained from circular RNA interactome database (Figure 4A). Then, we conducted the luciferase activity assay to confirm the binding relationship between hsa_circ_0128846 and miR‐1184, finding that miR‐1184 obviously reduced the luciferase activity in cells transfected with wild‐type circ_0128846 but not those transfected with mutated circ_0128846 (Figure 4B). In addition, RIP analysis revealed that hsa_circ_0128846 was utterly pulled down when transfected with miR‐1184 mimics in SW480 and HCT116 cells, compared to the miR‐1184 NC group (Figure 4C). Interestingly, miR‐1184 was found down‐regulated in CRC tissues compared with adjacent healthy tissues (Figure 4D), and there was a negative relationship between miR‐1184 and hsa_circ_0128846 expression (Figure 4E). Thus, we concluded that miR‐1184 was a downstream target of hsa_circ_0128846. Before we conducted further experiments to study the effects of hsa_circ_0128846 and miR‐1184, we examined the transfection efficiency of sh‐circ_0128846 and miR‐1184 inhibitor in SW480 and HCT116 cells. We validated that sh‐circ_0128846 could significantly down‐regulate the expression of circ_0128846 and up‐regulate that of miR‐1184. miR‐1184 inhibitor could significantly down‐regulate the expression of miR‐1184 but had no effects on circ_0128846 expression. sh‐circ_0128846 could also reverse the down‐regulation effects of miR‐1184 inhibitor in both HCT116 and SW480 cells caused by miR‐1184 inhibitor (Figure 4F).

### 3.5 Knockdown of hsa_circ_0128846 inhibited the proliferation, migration and invasion and promoted the apoptosis of CRC cells in vitro by sponging miR‐1184

**Figure 5:**
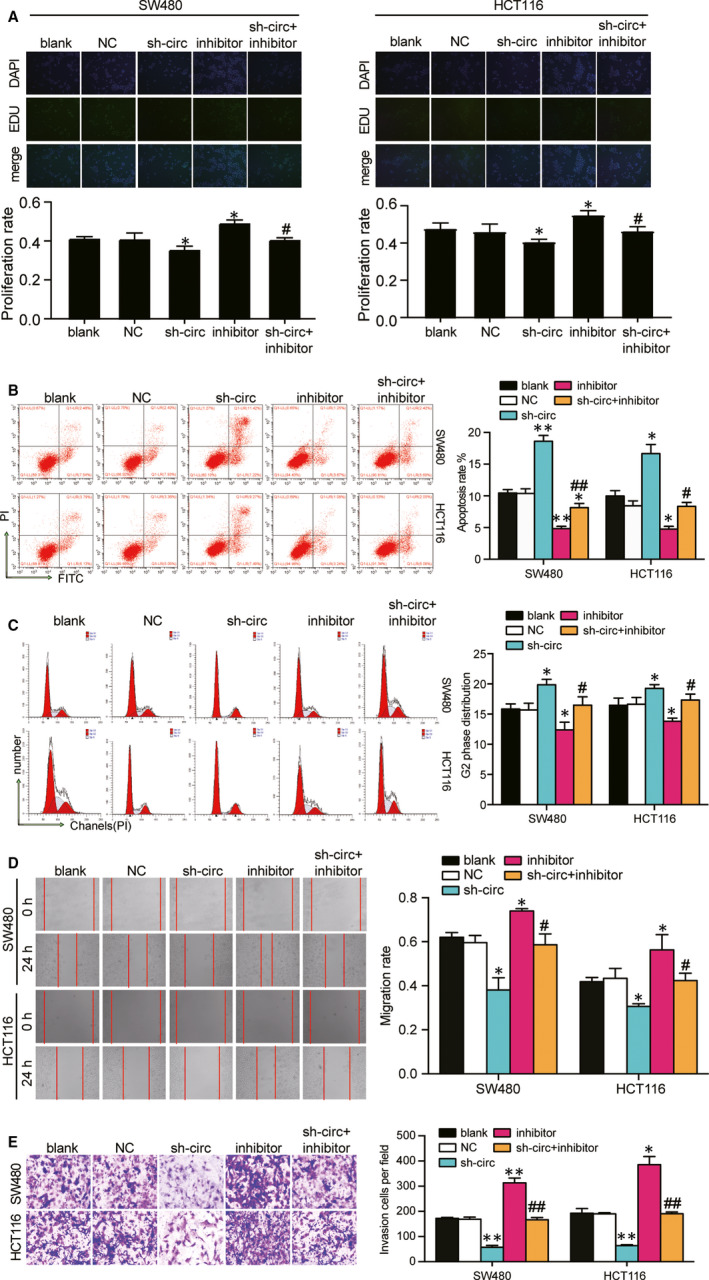
Effect of circ 0 128 846 on the development of CRC cells via sponging miR‐1184. (A) EdU assay was used to assess the proliferation of the SW480 and HCT116 cells with different transfection. The merged images were from EdU staining (green) and DAPI (blue) for SW480 and HCT116 cells. (B) The cell apoptosis was detected by flow cytometry in SW480 and HCT116 with different transfections. UR, the late apoptosis. LR, the early apoptosis. The cell apoptosis was the sum of the UR and LR. (C) Flow cytometry was performed to detect the cell cycle progression in the transfected SW480 and HCT116 cells. (D) The wound healing assay was used to assess the migration ability of SW480 and HCT116 cells after transfection at 0 and 24 hours. (E) The number of invaded cells was identified by transwell invasion assay. Blank, the cells were cultured without any treatments. NC, the cells were transfected with negative control plasmids. sh‐circ, the cells were transfected with circ_0128846 shRNA plasmids. Inhibitor, the cells were transfected with miR‐1184 inhibitor plasmids. sh‐circ + inhibitor, the cells were co‐transfected with circ_0128846 shRNA and miR‐1184 inhibitor plasmids. *P < .05, **P < .001 versus blank group. #*P* < .05, ##*P* < .001 versus sh‐circ group

To further explore whether hsa_circ_0128846 could affect the phenotypes of CRC cells via sponging miR‐1184, we performed the rescue experiments. The EdU assay showed that the co‐transfection of sh‐circ_0128846 and miR‐1184 inhibitor in SW480 and HCT116 cells led to a 1.14‐fold increase of proliferation rate compared with the transfection of sh‐circ_0128846 alone (Figure 5A). As shown in Figure 5B, miR‐1184 inhibitor decreased the apoptosis rate of HCT116 cells and SW480 cells, but co‐transfecting sh‐circ_0128846 and miR‐1184 inhibitor reversed the effect of miR‐1184 inhibitor on apoptosis rate of CRC cells. Additionally, FACS analysis showed that the co‐transfection of miR‐1184 relieved the cell cycle arrest at G2 phase that was induced by sh‐circ_0128846 in HCT116 cells and SW480 cells (Figure 5C). Moreover, it was discovered that miR‐1184 inhibitor obviously increased the cell migration rate by 1.2‐fold in SW480 cells and 1.3‐fold in HCT116 cells, while the co‐transfection group was not different from the blank group in the two CRC cell lines (Figure 5D). The transwell invasion assay showed that the transfection of miR‐1184 inhibitor led to a 1.8‐fold increase of the number of invaded cells in SW480 cells and a 2‐fold increase of that in HCT116 cells, while the transfection of sh‐circ_0128846 reversed the pro‐invading effect induced by miR‐1184 inhibitor in HCT116 cells and SW480 cells (Figure 5E). Taken together, the knockdown of hsa_circ_0128846 inhibited the phenotypes of CRC cells by freeing miR‐1184 in vitro.

### 3.6 AJUBA was a downstream target of miR‐1184

**Figure 6:**
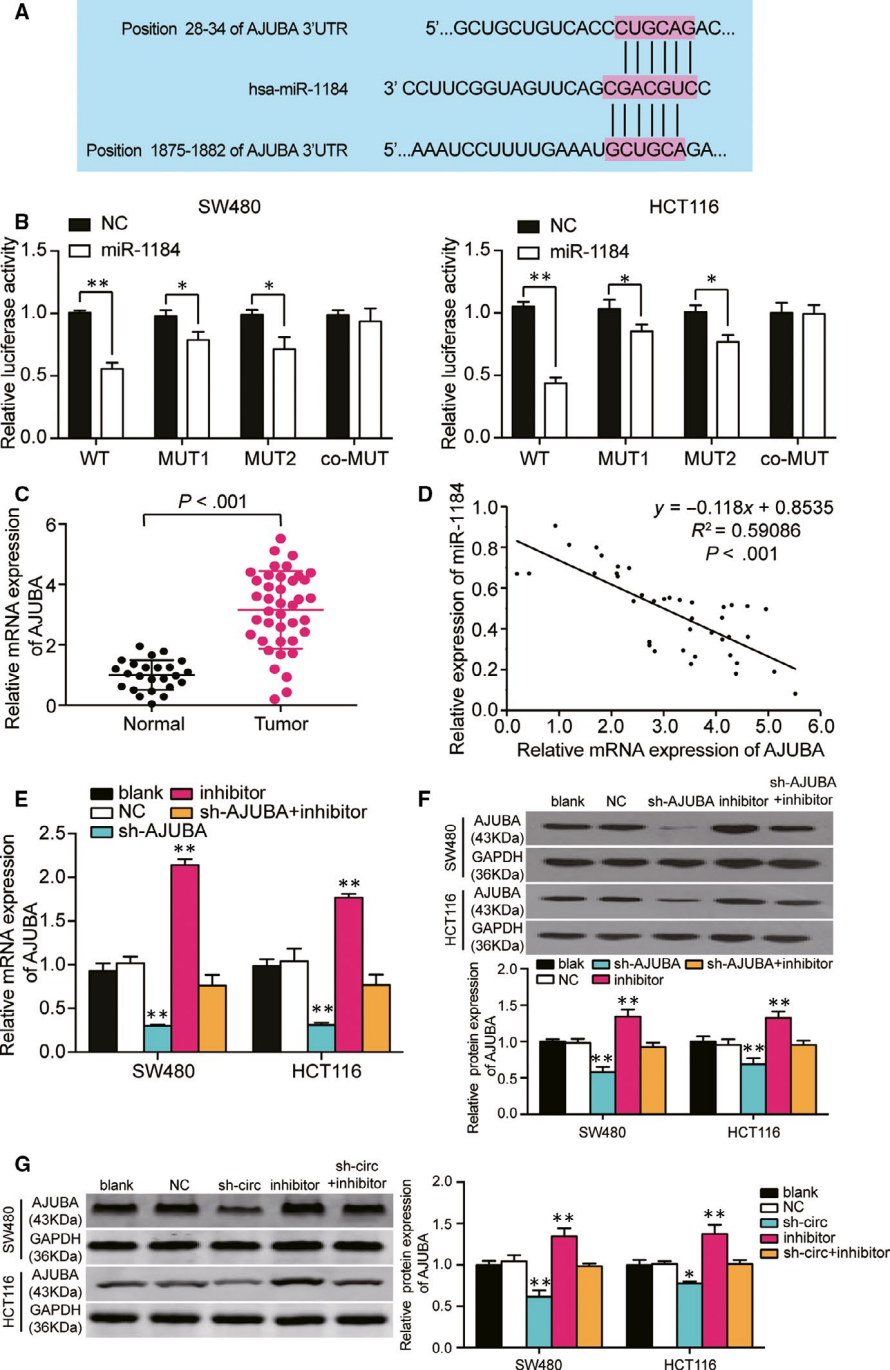
AJUBA was a target gene of miR‐1184, and regulated by hsa_circ_0128846. (A) The TargetScan Human 7.2 predicted the two binding sites between AJUBA 3′UTR and miR‐1184. (B) The luciferase reporter assay was used to validate the binding sites of AJUBA 3′UTR and miR‐1184 in SW480 and HCT116 cells. *P < .05 versus the miR‐NC group. (C) The expression of AJUBA was up‐regulated in human tumour tissue samples. (D) AJUBA expression was negatively correlated with miR‐1184 expression in CRC tumour tissues. (E‐F) qRT‐PCR and Western bot assay were employed to determine the expression of AJUBA mRNA and protein in the SW480 and HCT116 cells with different transfections. Blank, the cells were cultured without any treatments. NC, the cells were transfected with negative control plasmids. sh‐AJUBA, the cells were transfected with AJUBA shRNA plasmids. Inhibitor, the cells were transfected with miR‐1184 inhibitor plasmids. sh‐AJUBA + inhibitor, the cells were co‐transfected with AJUBA shRNA and miR‐1184 inhibitor plasmids. *P < .05, **P < .001 versus blank group. (G) The expression of AJUBA protein was regulated by hsa_circ_0128846. Blank, the cells were cultured without any treatments. NC, the cells were transfected with negative control plasmids. sh‐circ, the cells were transfected with circ_0128846 shRNA plasmids. Inhibitor, the cells were transfected with miR‐1184 inhibitor plasmids. sh‐circ + inhibitor, the cells were co‐transfected with circ_0128846 shRNA and miR‐1184 inhibitor plasmids. *P < .05, **P < .001 versus blank group

AJUBA was identified from the intersection of differentially expressed mRNAs of GSE126095 data set and the Hippo signalling pathway genes. Among the nine common mRNAs, AJUBA and SCRIB were the upstream effectors of the Hippo signalling pathway, whereas the others were all the downstream effectors (Fig. S1B). Then, we interrogated TCGA database and found that SCIB was not significantly up‐regulated in colon adenocarcinoma, but AJUBA was (Fig. S1C). TargetScan Human 7.2 was used to predict the binding relationship between AJUBA mRNA and miR‐1184 (Figure 6A). The luciferase activity in the wild‐type AJUBA + miR‐1184 group was the lowest, and that in the mutated sites 1 and 2 of AJUBA 3′UTR groups was lower than that in the co‐mutated AJUBA 3′UTR group (Figure 6B). Interestingly, we found that AJUBA showed a 3‐fold up‐regulation in tumour tissues compared with adjacent healthy tissues (Figure 6C) and a negative correlation with the expression of miR‐1184 in tumour tissues (Figure 6D). These results proved that AJUBA was a downstream target of miR‐1184. Before further experiments on miR‐1184 and AJUBA, we examined the transfection efficiency of three shRNAs of AJUBA and miR‐1184 inhibitor and found that shRNA1‐AJUBA induced the lowest expression of AJUBA in SW480 and HCT116 (Fig. S3). Then, we transfected shRNA1‐AJUBA (sh‐AJUBA) and miR‐1184 inhibitor into HCT116 cells and SW480 cells. The data revealed that sh‐AJUBA inhibited the expression of AJUBA by almost 70% in CRC cells, miR‐1184 inhibitor unregulated the expression of AJUBA by 2.1‐fold in SW480 cells and 1.8‐fold in HCT116 cells, and the expression of AJUBA in co‐transfection group did not show any difference from the blank group (Figure 6E). The expression of AJUBA protein displayed a similar trend as the expression of AJUBA mRNA (Figure 6F). Besides, we also detected the expression of AJUBA protein in the CRC cells transfected with sh‐circ_0128846 and/or miR‐1184 inhibitor, finding that sh‐circ_0128846 resulted in about a 30% decrease of the expression of AJUBA protein in CRC cells, and the expression of AJUBA protein in the co‐transfection group was similar to that in the blank group (Figure 6G).

### 3.7 The knockdown of miR‐1184 enhanced the phenotypes of CRC cells by targeting AJUBA thus further affecting Hippo/YAP signalling pathway

**Figure 7:**
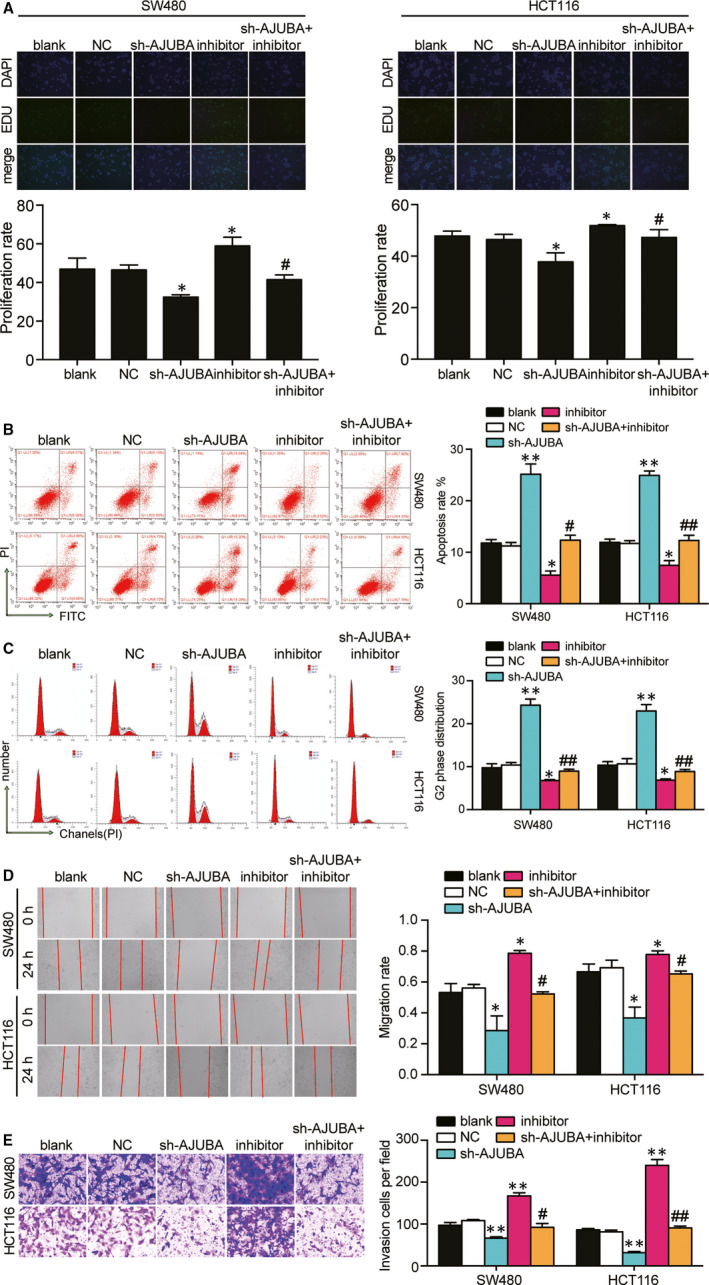
Effect of miR‐1184 on cell proliferation, cell apoptosis, cell cycle, cell migration and cell invasion in CRC cells via targeting AJUBA. (A) EdU assay was used to assess the cell proliferation capability in the transfected SW480 and HCT116 cells. The merged images were from EdU staining (green) and DAPI (blue) for SW480 and HCT116 cells. (B) The cell apoptosis was detected by flow cytometry in SW480 and HCT116 after transfection of different plasmids. UR, the late apoptosis. LR, the early apoptosis. The cell apoptosis was the sum of the UR and LR. (C) The cell cycle distribution was analysed by flow cytometry in the transfected SW480 and HCT116 cells. (D) The wound healing assay was used to assess the migration ability of SW480 and HCT116 cells after transfection at 0 and 24 hours. (E) The number of invaded cells was identified by transwell invasion assay. Blank, the cells were cultured without any treatments. NC, the cells were transfected with negative control plasmids. sh‐AJUBA, the cells were transfected with AJUBA shRNA plasmids. Inhibitor, the cells were transfected with miR‐1184 inhibitor plasmids. sh‐AJUBA + inhibitor, the cells were co‐transfected with AJUBA shRNA and miR‐1184 inhibitor plasmids. *P < .05, **P < .001 versus blank group. #*P* < .05, ##*P* < .001 versus sh‐AJUBA group

**Figure 8:**
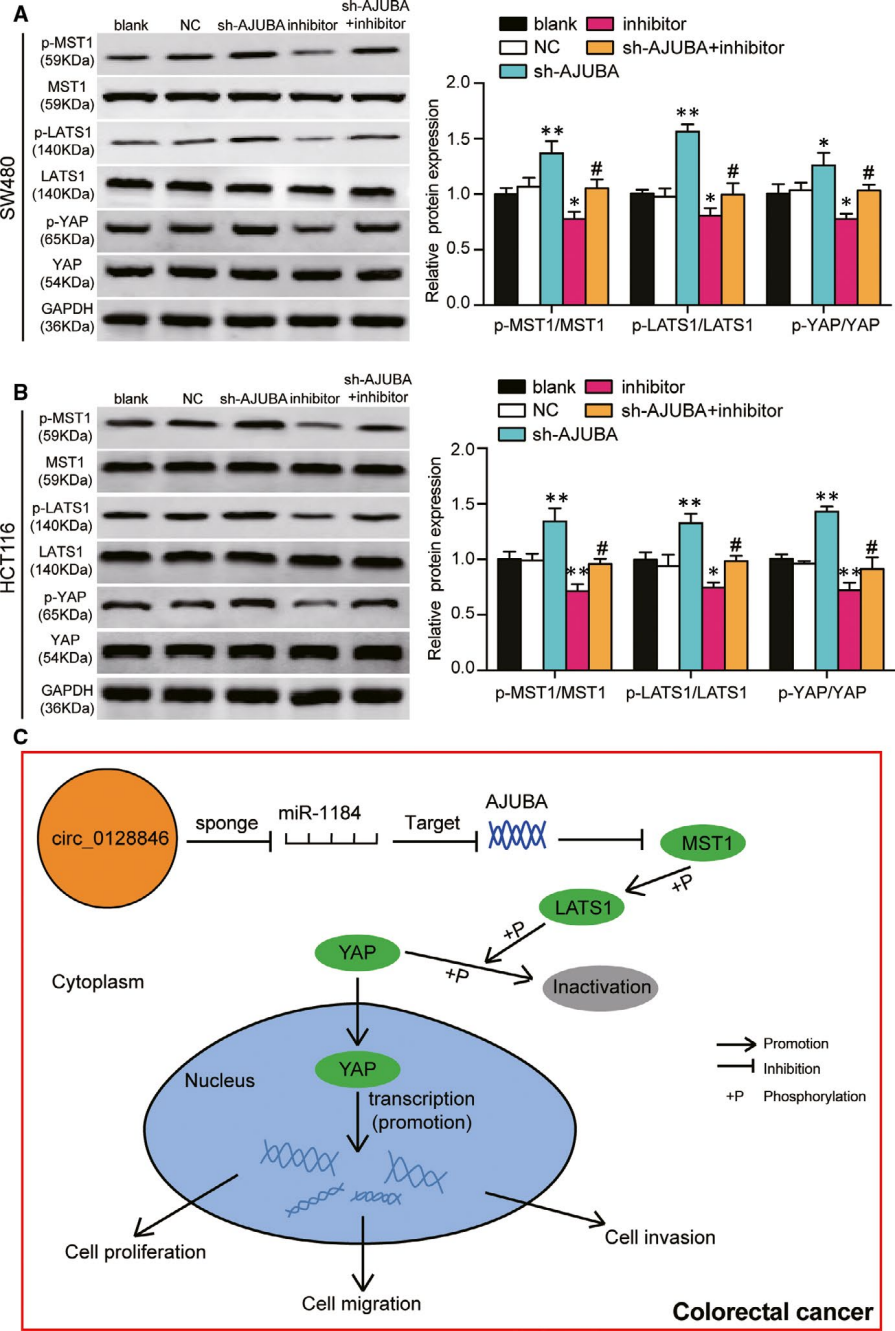
The miR‐1184/AJUBA axis could affect the Hippo/YAP signalling pathway. (A) The expression of proteins involving in the Hippo/YAP signalling pathway was measured by Western blot assay in SW480 cells that were transfected with the different plasmids. MST1, LATS1 and YAP were the key proteins in the Hippo/YAP signalling pathway. p‐MST1, the phosphorylated MST1 protein. p‐LATS1, the phosphorylated LATS1 protein. p‐YAP, the phosphorylated YAP protein. Blank, the cells were cultured without any treatments. NC, the cells were transfected with negative control plasmids. sh‐AJUBA, the cells were transfected with AJUBA shRNA plasmids. Inhibitor, the cells were transfected with miR‐1184 inhibitor plasmids. sh‐AJUBA + inhibitor, the cells were co‐transfected with AJUBA shRNA and miR‐1184 inhibitor plasmids. *P < .05, **P < .001 versus blank group. #*P* < .05, ##*P* < .001 versus sh‐AJUBA group. (B) The expression of proteins involving in the Hippo/YAP signalling pathway was measured by Western blot assay in HCT116 cells that were transfected with the different plasmids. MST1, LATS1 and YAP were the key proteins of the Hippo/YAP signalling pathway. p‐MST1, the phosphorylated MST1 protein. p‐LATS1, the phosphorylated LATS1 protein. p‐YAP, the phosphorylated YAP protein. Blank, the cells were cultured without any treatments. NC, the cells were transfected with negative control plasmids. sh‐AJUBA, the cells were transfected with AJUBA shRNA plasmids. Inhibitor, the cells were transfected with miR‐1184 inhibitor plasmids. sh‐AJUBA + inhibitor, the cells were co‐transfected with AJUBA shRNA and miR‐1184 inhibitor plasmids. *P < .05, **P < .001 versus blank group. #*P* < .05, ##*P* < .001 versus sh‐AJUBA group. (C) The potential mechanism of hsa_circ_0128846/miR‐1184/AJUBA axis on the progression of CRC

First of all, EdU assay was conducted to study the role of miR‐1184 and AJUBA in CRC cell proliferation. It was showed that AJUBA knockdown significantly suppressed the proliferating of both cell lines by approximately a third, and the co‐transfection of sh‐AJUBA with miR‐1184 inhibitor increased the proliferation rate compared with sh‐AJUBA (41.4% versus 32.4%) group in SW480 cells, and a similar trend was also observed in HCT116 cells (Figure 7A). The cell apoptosis assay displayed that the knockdown of AJUBA significantly resulted in the increased cell apoptosis (approximately 2‐fold of the blank group), and the apoptosis rate in the co‐transfection group decreased by 51% in SW480 and HCT116 cells compared with sh‐AJUBA group (Figure 7B). At the same time, the knockdown of AJUBA led to a significant G2 phase arrest ratio (approximately 2‐fold of the blank group), and the co‐transfection of sh‐AJUBA and miR‐1184 inhibitor relieved the cell cycle arrest at G2 phase induced by sh‐AJUBA (Figure 7C). What’ more, wound healing assay and transwell assay also discovered that the suppressing effect of sh‐AJUBA on cell migration and invasion was relieved by the co‐transfection with miR‐1184 inhibitor in HCT116 cells and SW480 cells (Figure 7D and E). Lastly, as a critical member of Hippo/YAP signalling pathway, we wondered how miR‐1184’s regulating AJUBA affected Hippo/YAP signalling. The expression of pathway‐associated proteins, MST1, LATS1 and YAP, was detected. AJUBA knockdown led to a 1.38‐fold increase of the rate of phosphorylated MST1 protein, a 1.56‐fold increase of the rate of phosphorylated LATS1 protein and a 1.26‐fold increase of the rate of phosphorylated YAP protein compared with the blank group. Then, the effect of sh‐AJUBA on pathway was relieved by co‐transfection with miR‐1184 inhibitor in SW480 cells (Figure 8A), indicating that AJUBA knockdown led to the activation of Hippo/YAP signalling and miR‐1184 inhibition led to the inactivation of Hippo‐YAP signalling. The similar results also displayed in HCT116 cells (Figure 8B). These data suggested that miR‐1184 inhibitor enhanced the phenotypes of CRC cells via releasing AJUBA to inactivate the Hippo/YAP signalling pathway (a schematic mechanism illustration is presented in Figure 8C).

## 4 DISCUSSION

In our paper, in vitro and in vivo experiments demonstrated that the expression of hsa_circ_0128846 was raised in CRC tissues and CRC cells, as well, hsa_circ_0128846 contributed to CRC cell proliferation, migration, invasion and cell cycle progression while inhibited cell apoptosis. What's more, miR‐1184 reversed the CRC cell phenotype‐promoting effect of circ_0128846. Furthermore, we also found that the downstream target of miR‐1184, AJUBA, could inactivate the Hippo/YAP signalling pathway to promote the development of CRC.

Circular RNAs have been proved to be crucial in the development of human cancers including human CRC; for example, ciRS‐7‐A, hsa_circ_101555 and circNSD2 were once characterized to be significantly up‐regulated in CRC.^38^, ^39^, ^40^ It was reported that circ_0053277 with the high expression level in CRC tissues and cells enhanced cell viability, migration and epithelial‐mesenchymal transition process in CRC.^41^ In our research, we found that hsa_circ_0128846 was significantly up‐regulated in CRC tissues and cells, and it also contributed to enhanced cell proliferation, migration and invasion in vitro, as well as tumour growth in vivo. Silencing hsa_circ_0128846, on the other hand, led to the significant cell cycle arrest at G2 phase and the increase of cell apoptosis rate in CRC cells.

In addition, there were other studies reporting the mechanism of competitive endogenous RNA (ceRNA) networks that involve circRNAs and miRNAs in CRC tumour formation. For instance, Geng Y’s research suggested that hsa_circ_0009361 acted as a sponge of miR‐582 to impair the ability of the CRC tumorigenicity by regulating APC2 expression. In their research, hsa_circ_0009361 and APC2 were both reported to be up‐regulated, while miR‐582 was down‐regulated in CRC.^19^ In another research, hsa_circ_0136666 was able to up‐regulate SH2B1 expression through competitively binding with miR‐136 to promote CRC cell metastasis.^42^ Together, circular RNAs with affluent binding sites for miRNAs could act as miRNA sponges in tumour cells, thus affecting tumour progression. In our study, we also found that hsa_circ_0128846 had a binding site for miR‐1184 and down‐regulated the expression of miR‐1184, thereby promoting the development of CRC.

It is widely accepted that microRNA participates in the base pairing of the 3′UTR of the target gene, thereby halting the transcription or the translation process of the target.^43^, ^44^ MiR‐1184 was first reported by Chen G, *et al* ^25^ in 2016. Their team used GSE37178 data series from Gene Expression Omnibus database to identify differentially expressed genes and screen out the potential diagnostic or therapeutic target genes for CRC. They found that the expression of miR‐1184 was significantly reduced in CRC compared with the healthy control. In our study, we found that the expression of miR‐1184 was down‐regulated in CRC tissues and cells by qRT‐PCR which was the same with Chen, *et al*’s. It was once reported that miR‐1184 could increase insulin‐like growth factor‐binding protein 2, thereby promoting the aggressive biological behaviours of bladder cancer cells.^20^ In addition, there was a positive relationship between miR‐1184 and PD‐L1 mRNA, suggesting that miR‐1184 might regulate PD‐L1 expression at a post‐transcription level in non‐small‐cell lung cancer.^21^ Similarly, miR‐1184 in our study was proved to inhibit the CRC cell phenotypes via suppressing AJUBA expression. Taking together, we concluded that miR‐1184 was a significant tumour suppressor in human CRC.

AJUBA protein, consisting of 55 amino acids, is a member of the Zyxin/AJUBA family, which also includes Zyxin, Trip6, Wtip6, Limd1 and Lpp.^28^, ^45^, ^46^, ^47^, ^48^ AJUBA is composed of the carboxy‐terminal LIM region and the amino‐terminal PreLIM region,^27^, ^48^, ^49^ and is a cytoplasmic linker protein that had the ability to shuttle between the cell junction and the nucleus, thereby playing an important part in signal transmission.^50^ AJUBA was reported to be an upstream regulator of the Hippo signalling pathway,^51^ and it had the function of an epithelial integrity sensor and was necessary for re‐entering the cell cycle progression.^31^, ^48^ In addition, AJUBA promoted oncogenic activity of YES‐associated protein (YAP) by suppressing large tumour suppressor type 1/2 (LATS 1/2) core kinase in the Hippo signalling pathway.^29^, ^31^, ^52^, ^53^ In other words, AJUBA could interact with LATS to prevent LATS from phosphorylating YAP, which was active in the un‐phosphorylated state to increase cell proliferation.^29^, ^31^, ^52^, ^53^ What's more, Song *et al* ^54^ proved that the expression of YAP in CRC patients was high which was associated with an additional risk of cancer recrudesce and poor outcome of CRC patients who underwent 5‐FU treatment. Above all, we speculated that the knockdown of AJUBA could increase YAP phosphorylation to promote the activation of Hippo/YAP signalling pathway, thereby suppressing cell proliferation and migration of CRC cells. This speculation was confirmed by our results that the knockdown of AJUBA increased the phosphorylated MST1, phosphorylated LATS1 and phosphorylated YAP, thereby inactivating Hippo/YAP signalling pathway. However, the effect of AJUBA knockdown could be reversed by miR‐1184 inhibitor.

There are deficiencies in our study. Although we confirmed that the knockdown of AJUBA inactivated the Hippo/YAP signalling pathway, we did not study in depth how AJUBA protein entered the nucleus and interacted with YAP protein. What's more, tumour metastasis was a critical biological feature of malignant tumours and the most fatal attribute of cancer. The quality of life and standard of living of the patient who undergoes metastasis significantly decreases. Therefore, inhibiting CRC metastasis was considered to be an efficacious method to improve the prognosis of patient. In our research, we did not study the metastasis in nude mice due to the experimental limitations. Future researches would be conducted to address these issues.

In summary, our study suggests that circ_0128846 promotes the development of CRC and inactivates Hippo/YAP signalling pathway by decreasing the expression of miR‐1184, thereby increasing AJUBA expression.

## CONFLICT OF INTERESTS

None declared by the authors.

## AUTHOR CONTRIBUTION

**Xu Wang:** Methodology (equal); Project administration (equal). **Yujia Chen:** Methodology (equal); Project administration (equal). **Wei Liu:** Data curation (lead); Writing‐original draft (lead); Writing‐review & editing (equal). **Tao Liu:** Conceptualization (equal); Investigation (equal). **Di Sun:** Conceptualization (equal); Investigation (equal); Writing‐review & editing (equal).

## AUTHOR CONTRIBUTION

TL and DS designed the study. XW and YJC performed the experiments. WL interpreted the data and drafted the manuscript. All authors reviewed and edited the manuscript.

## Supporting information


**Figure S1**.

**Figure S2**.

**Figure S3**.

## Data Availability

The data used in this study are available from the corresponding author upon reasonable request.

## References

[1] Bray F , Ferlay J , Soerjomataram I , Siegel RL , Torre LA , Jemal A . Global cancer statistics 2018: GLOBOCAN estimates of incidence and mortality worldwide for 36 cancers in 185 countries. CA Cancer J Clin. 2018;68(6):394‐424. 30207593 10.3322/caac.21492

[2] Kopetz S , Chang GJ , Overman MJ , et al. Improved survival in metastatic colorectal cancer is associated with adoption of hepatic resection and improved chemotherapy. J Clin Oncol. 2009;27(22):3677‐3683. 19470929 10.1200/JCO.2008.20.5278 PMC2720081

[3] Chen WZ , Jiang JX , Yu XY , et al. Endothelial cells in colorectal cancer. World J Gastrointest Oncol. 2019;11(11):946‐956. 31798776 10.4251/wjgo.v11.i11.946 PMC6883186

[4] Hua CC . Associations between the Nrf2/Keap1 pathway and mitochondrial functions in colorectal cancer are affected by metastasis. Cancer Biomark. 2019; 27(2): 163‐171. 10.3233/CBM-190828 PMC12662278 31796664

[5] Iravani S , Eslami P , Dooghaie Moghadam A , et al. The role of melatonin in colorectal cancer. J Gastrointest Cancer. 2019 10.1007/s12029-019-00336-4.. [Epub ahead of print]. 31792737

[6] Sur D , Cainap C , Burz C , et al. The role of miRNA ‐31‐3p and miR‐31‐5p in the anti‐EGFR treatment efficacy of wild‐type K‐RAS metastatic colorectal cancer. Is it really the next best thing in miRNAs? J BUON. 2019;24(5):1739‐1746. 31786833

[7] Grassadonia A , Di Marino P , Ficorella C , et al. Impact of primary tumor location in patients with RAS wild‐type metastatic colon cancer treated with first‐line chemotherapy plus anti‐EGFR or anti‐VEGF monoclonal antibodies: a retrospective multicenter study. J Cancer. 2019;10(24):5926‐5934. 31762802 10.7150/jca.34550 PMC6856567

[8] Jung WB , Shin JY , Suh BJ . The short‐term outcome and safety of laparoscopic colorectal cancer resection in very elderly patients. Korean J Gastroenterol. 2017;69(5):291‐297. 28539034 10.4166/kjg.2017.69.5.291

[9] Joosen AM , Kuhnle GG , Aspinall SM , et al. Effect of processed and red meat on endogenous nitrosation and DNA damage. Carcinogenesis. 2009;30(8):1402‐1407. 19498009 10.1093/carcin/bgp130 PMC2718076

[10] Morabito A , De Maio E , Di Maio M , Normanno N , Perrone F . Tyrosine kinase inhibitors of vascular endothelial growth factor receptors in clinical trials: current status and future directions. Oncologist. 2006;11(7):753‐764. 16880234 10.1634/theoncologist.11-7-753

[11] Qu S , Zhong Y , Shang R , et al. The emerging landscape of circular RNA in life processes. RNA Biol. 2017;14(8):992‐999. 27617908 10.1080/15476286.2016.1220473 PMC5680710

[12] Qu S , Yang X , Li X , et al. Circular RNA: A new star of noncoding RNAs. Cancer Lett. 2015;365(2):141‐148. 26052092 10.1016/j.canlet.2015.06.003

[13] Cortes‐Lopez M , Miura P . Emerging functions of circular RNAs. Yale J Biol Med. 2016;89(4):527‐537. 28018143 PMC5168830

[14] Barrett SP , Salzman J . Circular RNAs: analysis, expression and potential functions. Development. 2016;143(11):1838‐1847. 27246710 10.1242/dev.128074 PMC4920157

[15] Hansen TB , Kjems J , Damgaard CK . Circular RNA and miR‐7 in cancer. Cancer Res. 2013;73(18):5609‐5612. 24014594 10.1158/0008-5472.CAN-13-1568

[16] Zheng Q , Bao C , Guo W , et al. Circular RNA profiling reveals an abundant circHIPK3 that regulates cell growth by sponging multiple miRNAs. Nat Commun. 2016;7:11215. 27050392 10.1038/ncomms11215 PMC4823868

[17] Zeng K , Chen X , Xu M , et al. CircHIPK3 promotes colorectal cancer growth and metastasis by sponging miR‐7. Cell Death Dis. 2018;9(4):417. 29549306 10.1038/s41419-018-0454-8 PMC5856798

[18] Li X , Wang J , Zhang C , et al. Circular RNA circITGA7 inhibits colorectal cancer growth and metastasis by modulating the Ras pathway and upregulating transcription of its host gene ITGA7. J Pathol. 2018;246(2):166‐179. 29943828 10.1002/path.5125

[19] Geng Y , Zheng X , Hu W , et al. Hsa_circ_0009361 acts as the sponge of miR‐582 to suppress colorectal cancer progression by regulating APC2 expression. Clin Sci (Lond). 2019;133(10):1197‐1213. 31109967 10.1042/CS20190286

[20] Yang D , Qian H , Fang Z , et al. Silencing circular RNA VANGL1 inhibits progression of bladder cancer by regulating miR‐1184/IGFBP2 axis. Cancer Med. 2020;9(2):700‐710. 31758655 10.1002/cam4.2650 PMC6970048

[21] Grenda A , Nicos M , Szczyrek M , et al. MicroRNAs aid the assessment of programmed death ligand 1 expression in patients with non‐small cell lung cancer. Oncol Lett. 2019;17(6):5193‐5200. 31186735 10.3892/ol.2019.10207 PMC6507482

[22] Danza K , De Summa S , Pinto R , et al. TGFbeta and miRNA regulation in familial and sporadic breast cancer. Oncotarget. 2017;8(31):50715‐50723. 28881597 10.18632/oncotarget.14899 PMC5584195

[23] Feng F , Wu J , Gao Z , Yu S , Cui Y . Screening the key microRNAs and transcription factors in prostate cancer based on microRNA functional synergistic relationships. Medicine (Baltimore). 2017;96(1):e5679. 28072703 10.1097/MD.0000000000005679 PMC5228663

[24] Knyazev EN , Fomicheva KA , Mikhailenko DS , et al. Plasma levels of hsa‐miR‐619‐5p and hsa‐miR‐1184 differ in prostatic benign hyperplasia and cancer. Bull Exp Biol Med. 2016;161(1):108‐111. 27265125 10.1007/s10517-016-3357-7

[25] Chen G , Han N , Li G , et al. Time course analysis based on gene expression profile and identification of target molecules for colorectal cancer. Cancer Cell Int. 2016;16:22. 27013928 10.1186/s12935-016-0296-3 PMC4806509

[26] Xu B , Li Q , Chen N , et al. The LIM protein Ajuba recruits DBC1 and CBP/p300 to acetylate ERalpha and enhances ERalpha target gene expression in breast cancer cells. Nucleic Acids Res. 2019;47(5):2322‐2335. 30597111 10.1093/nar/gky1306 PMC6412004

[27] Kanungo J , Pratt SJ , Marie H , Longmore GD . Ajuba, a cytosolic LIM protein, shuttles into the nucleus and affects embryonal cell proliferation and fate decisions. Mol Biol Cell. 2000;11(10):3299‐3313. 11029037 10.1091/mbc.11.10.3299 PMC14993

[28] Pratt SJ , Epple H , Ward M , Feng Y , Braga VM , Longmore GD . The LIM protein Ajuba influences p130Cas localization and Rac1 activity during cell migration. J Cell Biol. 2005;168(5):813‐824. 15728191 10.1083/jcb.200406083 PMC2171823

[29] Tanaka I , Osada H , Fujii M , et al. LIM‐domain protein AJUBA suppresses malignant mesothelioma cell proliferation via Hippo signaling cascade. Oncogene. 2015;34(1):73‐83. 24336325 10.1038/onc.2013.528

[30] Jia H , Song L , Cong Q , et al. The LIM protein AJUBA promotes colorectal cancer cell survival through suppression of JAK1/STAT1/IFIT2 network. Oncogene. 2017;36(19):2655‐2666. 27893714 10.1038/onc.2016.418

[31] Das Thakur M , Feng Y , Jagannathan R , Seppa MJ , Skeath JB , Longmore GD . Ajuba LIM proteins are negative regulators of the Hippo signaling pathway. Curr Biol. 2010;20(7):657‐662. 20303269 10.1016/j.cub.2010.02.035 PMC2855397

[32] Yang D , Hou T , Li L , et al. Smad1 promotes colorectal cancer cell migration through Ajuba transactivation. Oncotarget. 2017;8(66):110415‐110425. 29299158 10.18632/oncotarget.22780 PMC5746393

[33] Haraguchi K , Ohsugi M , Abe Y , Semba K , Akiyama T , Yamamoto T . Ajuba negatively regulates the Wnt signaling pathway by promoting GSK‐3beta‐mediated phosphorylation of beta‐catenin. Oncogene. 2008;27(3):274‐284. 17621269 10.1038/sj.onc.1210644

[34] Song K , Su W , Liu Y , et al. Identification of genes with universally upregulated or downregulated expressions in colorectal cancer. J Gastroenterol Hepatol. 2019;34(5):880‐889. 30395690 10.1111/jgh.14529

[35] Jia L , Gui B , Zheng D , et al. Androgen receptor‐regulated miRNA‐193a‐3p targets AJUBA to promote prostate cancer cell migration. Prostate. 2017;77(9):1000‐1011. 28422308 10.1002/pros.23356

[36] Choi KH , Shin CH , Lee WJ , Ji H , Kim HH . Dual‐strand tumor suppressor miR‐193b‐3p and ‐5p inhibit malignant phenotypes of lung cancer by suppressing their common targets. Biosci Rep. 2019;39(7):BSR20190634. 31262974 10.1042/BSR20190634 PMC6630026

[37] Song H , Yang PC . Construction of shRNA lentiviral vector. N Am J Med Sci. 2010;2(12):598‐601. 22558575 10.4297/najms.2010.2598 PMC3338230

[38] Weng W , Wei Q , Toden S , et al. Circular RNA ciRS‐7‐A promising prognostic biomarker and a potential therapeutic target in colorectal cancer. Clin Cancer Res. 2017;23(14):3918‐3928. 28174233 10.1158/1078-0432.CCR-16-2541 PMC5511556

[39] Huang ZM , Ge HF , Yang CC , et al. MicroRNA‐26a‐5p inhibits breast cancer cell growth by suppressing RNF6 expression. Kaohsiung J Med Sci. 2019;35(8):467‐473. 31063232 10.1002/kjm2.12085 PMC11900708

[40] Chen LY , Zhi Z , Wang L , et al. NSD2 circular RNA promotes metastasis of colorectal cancer by targeting miR‐199b‐5p‐mediated DDR1 and JAG1 signalling. J Pathol. 2019;248(1):103‐115. 30666650 10.1002/path.5238

[41] Xiao H , Liu M . Circular RNA hsa_circ_0053277 promotes the development of colorectal cancer by upregulating matrix metallopeptidase 14 via miR‐2467‐3p sequestration. J Cell Physiol. 2020;235(3):2881‐2890. 31549406 10.1002/jcp.29193

[42] Jin C , Wang A , Liu L , Wang G , Li G . Hsa_circ_0136666 promotes the proliferation and invasion of colorectal cancer through miR‐136/SH2B1 axis. J Cell Physiol. 2019;234(5):7247‐7256. 30370521 10.1002/jcp.27482

[43] Li Z , Xu R , Li N . MicroRNAs from plants to animals, do they define a new messenger for communication? Nutr Metab (Lond). 2018;15:68. 30302122 10.1186/s12986-018-0305-8 PMC6167836

[44] Reinhart BJ , Weinstein EG , Rhoades MW , Bartel B , Bartel DP . MicroRNAs in plants. Genes Dev. 2002;16(13):1616‐1626. 12101121 10.1101/gad.1004402 PMC186362

[45] Gaspar P , Holder MV , Aerne BL , Janody F , Tapon N . Zyxin antagonizes the FERM protein expanded to couple F‐actin and Yorkie‐dependent organ growth. Curr Biol. 2015;25(6):679‐689. 25728696 10.1016/j.cub.2015.01.010

[46] Rauskolb C , Pan G , Reddy BV , Oh H , Irvine KD . Zyxin links fat signaling to the hippo pathway. PLoS Biol. 2011;9(6):e1000624. 21666802 10.1371/journal.pbio.1000624 PMC3110180

[47] Bubenshchikova E , Ichimura K , Fukuyo Y , et al. Wtip and Vangl2 are required for mitotic spindle orientation and cloaca morphogenesis. Biol Open. 2012;1(6):588‐596. 23213452 10.1242/bio.20121016 PMC3509438

[48] Huggins CJ , Andrulis IL . Cell cycle regulated phosphorylation of LIMD1 in cell lines and expression in human breast cancers. Cancer Lett. 2008;267(1):55‐66. 18439753 10.1016/j.canlet.2008.03.015

[49] Feng Y , Zhao H , Luderer HF , et al. The LIM protein, Limd1, regulates AP‐1 activation through an interaction with Traf6 to influence osteoclast development. J Biol Chem. 2007;282(1):39‐48. 17092936 10.1074/jbc.M607399200

[50] Feng Y , Longmore GD . The LIM protein Ajuba influences interleukin‐1‐induced NF‐kappaB activation by affecting the assembly and activity of the protein kinase Czeta/p62/TRAF6 signaling complex. Mol Cell Biol. 2005;25(10):4010‐4022. 15870274 10.1128/MCB.25.10.4010-4022.2005 PMC1087715

[51] Rauskolb C , Sun S , Sun G , Pan Y , Irvine KD . Cytoskeletal tension inhibits Hippo signaling through an Ajuba‐Warts complex. Cell. 2014;158(1):143‐156. 24995985 10.1016/j.cell.2014.05.035 PMC4082802

[52] Abe Y , Ohsugi M , Haraguchi K , Fujimoto J , Yamamoto T . LATS2‐Ajuba complex regulates gamma‐tubulin recruitment to centrosomes and spindle organization during mitosis. FEBS Lett. 2006;580(3):782‐788. 16413547 10.1016/j.febslet.2005.12.096

[53] Rozengurt E , Sinnett‐Smith J , Eibl G . Yes‐associated protein (YAP) in pancreatic cancer: at the epicenter of a targetable signaling network associated with patient survival. Signal Transduct Target Ther. 2018;3:11. 29682330 10.1038/s41392-017-0005-2 PMC5908807

[54] Song R , Gu D , Zhang L , et al. Functional significance of Hippo/YAP signaling for drug resistance in colorectal cancer. Mol Carcinog. 2018;57(11):1608‐1615. 30074279 10.1002/mc.22883

