# Supplementary figures and images for: Hsa_circ_0128846 promotes tumorigenesis of colorectal cancer by sponging hsa‐miR‐1184 and releasing AJUBA and inactivating Hippo/YAP signalling
Source: J Cell Mol Med. 2020 Jul 18;24(17):9908–24. doi: 10.1111/jcmm.15590 (PMC7520282; doi:10.1111/jcmm.15590)

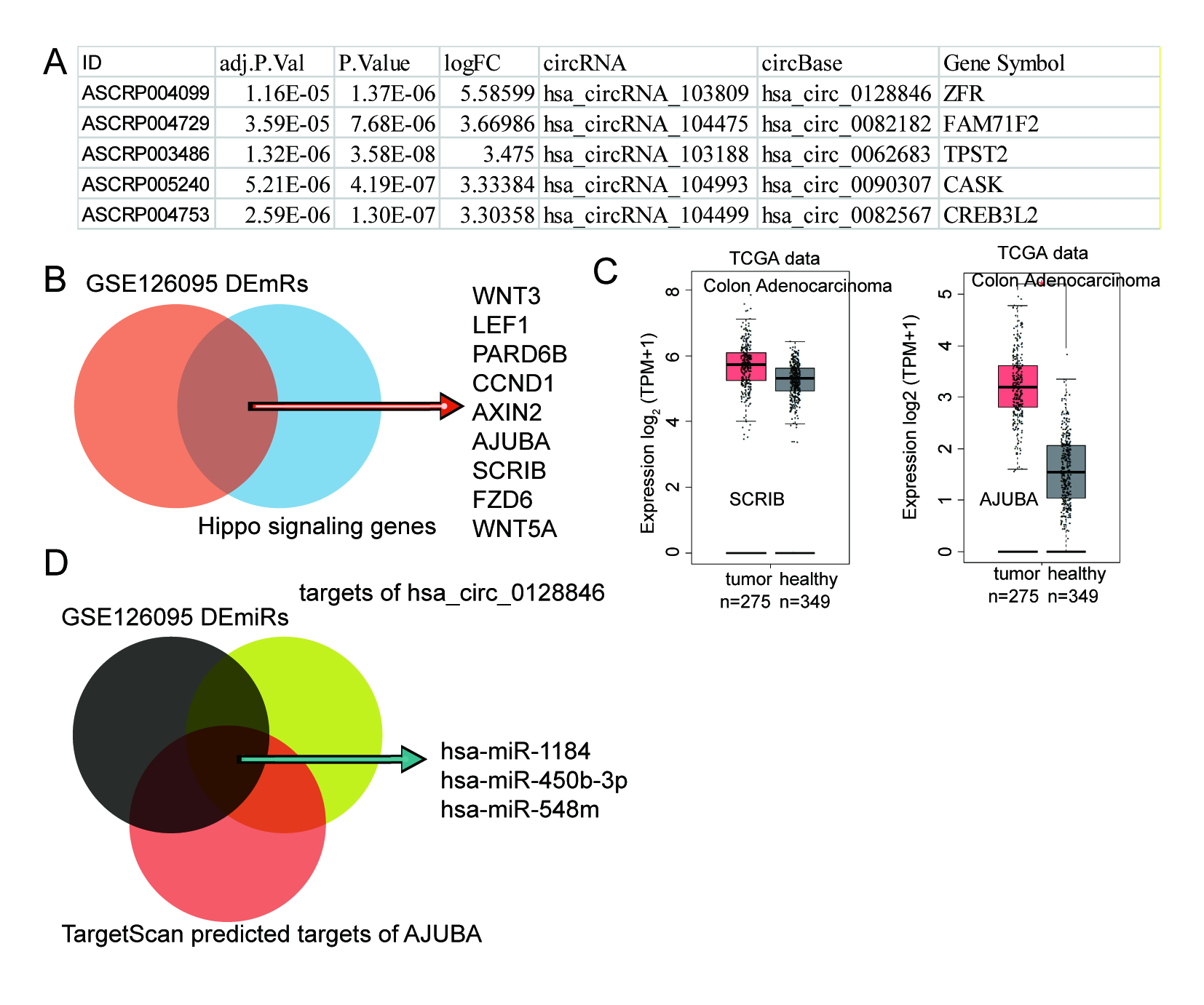

Supplement: Supplementary file 1 — Figure S1. [file JCMM-24-9908-s001.tif]

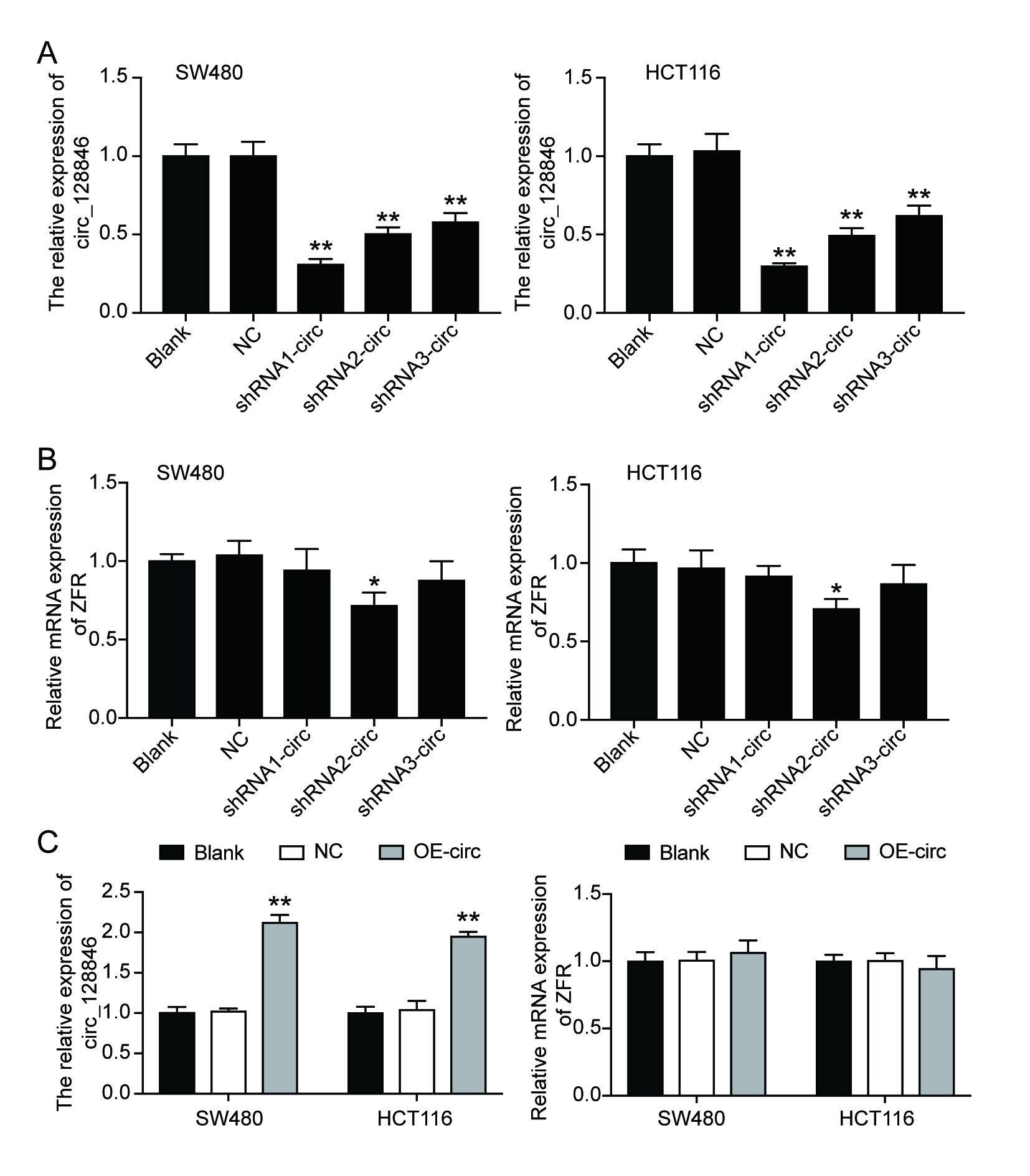

Supplement: Supplementary file 2 — Figure S2. [file JCMM-24-9908-s002.tif]

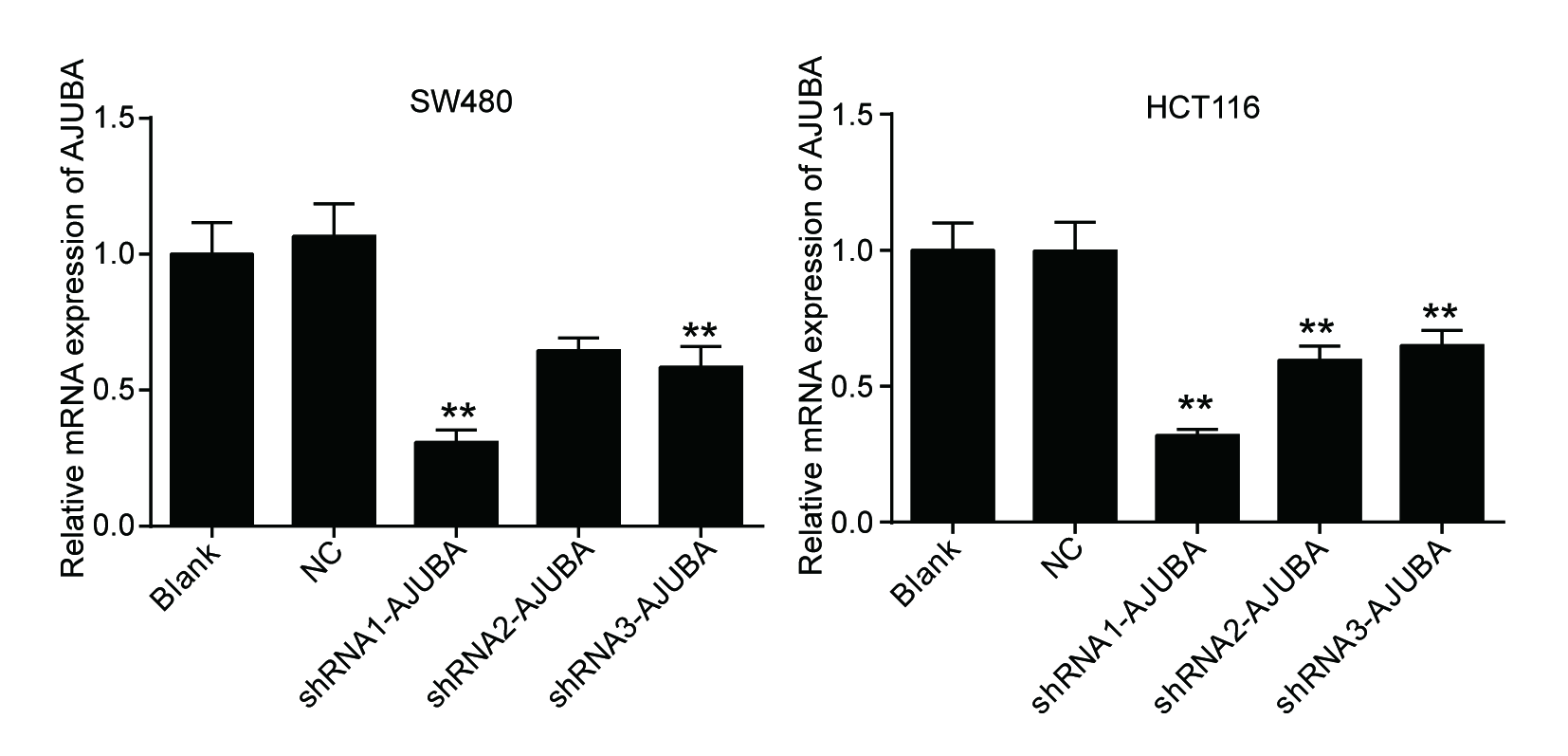

Supplement: Supplementary file 3 — Figure S3. [file JCMM-24-9908-s003.tif]
